# Chlorogenic acid modulates mitochondrial damage and mitophagy to repair injured myocardial tissue and cells

**DOI:** 10.3389/fphar.2025.1658090

**Published:** 2025-10-01

**Authors:** Hao Ling, Yu Zhang, Chunli Song

**Affiliations:** Department of General Practice and Special Ward, The Second Hospital of Jilin University, Changchun, China

**Keywords:** chlorogenic acid, mitochondrial damage, mitophagy, myocardial infarction, the PINK1/Parkin pathway

## Abstract

**Background:**

Chlorogenic acid (CGA) is a polyphenolic compound widely present in plants. It is primarily extracted from the leaves of Eucommia ulmoides, a native medicinal herb.

**Purpose:**

This study aimed to investigate the cardioprotective effects of CGA on myocardial infarction (MI) by modulating mitochondrial damage and mitophagy through the PINK1/Parkin signaling pathway.

**Methods:**

*In vitro*, H9C2 cardiomyocytes were treated with varying concentrations of CGA to assess cell viability, oxidative stress, mitochondrial function, and mitophagy markers (PINK1/Parkin) and mitophagic flux. *In vivo*, There are a total of 18 SD rats, which are randomly divided into Sham, MI, and CGA groups. MI was induced in SD rats via coronary artery ligation, followed by tail vein injection of CGA. Echocardiography, histological staining, electron microscopy, immunohistochemistry, Western blot (WB) and metabolomics analysis were conducted to evaluate cardiac function, tissue fibrosis, mitophagy, and metabolic changes.

**Results:**

CGA significantly improved cell viability and reduced oxidative stress in H9C2 cells. It also stabilized mitochondrial membrane potential, increased ATP levels, upregulated PINK1/Parkin expression and increase autophagy flux, Lysosomal inhibitor treatment elevates expression of Parkin, P62 and LC3-II. In MI rats, CGA reduced ROS levels, improved myocardial tissue integrity, reduced fibrosis, and enhanced cardiac function. Detected by electron microscopy, immunohistochemistry, and WB confirmed increased mitophagy in CGA-treated rats. Metabolomics analysis revealed significant alterations in metabolic pathways, particularly related to organic acids and amino acid metabolism.

**Conclusion:**

This study demonstrates that CGA exerts cardioprotective effects by modulating mitochondrial damage and promoting mitophagy through the PINK1/Parkin pathway. Our findings provide new insights into the therapeutic potential of CGA for MI treatment and suggest a promising natural compound for myocardial protection.

## 1 Introduction

Myocardial injury is a key pathological process in the progression of various cardiovascular diseases, closely related to energy metabolism disorders, reactive oxygen species (ROS) accumulation, and cell apoptosis induced by mitochondrial dysfunction (Bhimaraj and Young, 2024). Mitochondria, as crucial organelles for myocardial cell survival, when their homeostasis is imbalanced, can lead to mitochondrial membrane potential collapse, pro-apoptotic factor release, and a cascade of reactions, ultimately accelerating myocardial cell death (Ajoolabady et al., 2022; Ramachandra et al., 2020). Therefore, the selective removal of damaged mitochondria, i.e., promoting mitophagy, becomes an important regulatory mechanism for repairing myocardial cell injury and a critical step in myocardial infarction (MI) treatment (Liu et al., 2023).

The PINK1/Parkin pathway is the core molecular mechanism regulating mitophagy (Li et al., 2023). When the mitochondrial membrane potential depolarizes, PINK1 stabilizes and accumulates on the outer mitochondrial membrane, activating Parkin protein, which marks damaged mitochondria through ubiquitination, initiating the autophagolysosomal degradation process (Narendra and Youle, 2024). Studies have shown that moderate activation of this pathway effectively eliminates damaged mitochondria, restoring energy metabolism balance in myocardial cells (Cao et al., 2023). For example, gastrodin significantly reduces myocardial cell apoptosis and improves mitochondrial function by targeting the activation of the PINK1/Parkin pathway, suggesting the precise regulatory potential of natural compounds on this pathway (Chen et al., 2024).

Chlorogenic acid (CGA) is a polyphenolic compound widely present in plants. CGA is found in apples, cherries, blueberries, tomatoes, potatoes, hawthorn, and sunflower seeds. Sufficient amounts can be obtained from a healthy diet (Lu et al., 2020). The relationship between CGA and nutrition is reflected in multiple aspects. Research shows that CGA can alleviate myocardial cell damage induced by high glucose and high fat by reducing ROS generation and inhibiting mitochondrial permeability transition pore (mPTP) opening (Singh et al., 2023). Notably, the dynamic balance of mitochondrial membrane cardiolipin (CL) is closely related to the activation efficiency of the PINK1/Parkin pathway, and CGA may indirectly affect mitophagy by regulating CL metabolism (Hu et al., 2022). However, most existing studies focus on the antioxidant properties of CGA, and its direct regulatory effects on the PINK1/Parkin pathway and the mechanism in MI repair remain unclear. Meanwhile, traditional CGA can only be administered orally, but the bottleneck of low oral bioavailability (4%–8%) still needs to be addressed (Yu et al., 2022). Therefore, in this study, we attempted to improve the bioavailability of CGA in MI treatment through intravenous injection via the tail vein.

Based on the above background, this study focuses on the repair effects of CGA on mitochondrial damage in MI models *in vivo* and *in vitro*, emphasizing the molecular mechanism of its regulation of mitophagy through the PINK1/Parkin signaling pathway, providing new insights for the development of myocardial protection strategies targeting mitochondrial homeostasis.

## 2 Methods

### 2.1 Cell culture

DMEM medium (Gibco, USA) supplemented with 10% fetal bovine serum (FBS) (Gibco, USA) and 1% penicillin-streptomycin mixture was used. The H9C2 cell (Eoucell, China) were seeded in T25 flasks and grown adherently in a 37 °C, 5% CO_2_ incubator (Thermo Fisher Scientific, Waltham, MA, USA), with fresh medium replaced every 2–3 days. When cell density reached 80%–90%, cells were digested with 0.25% trypsin (Solarbio, China) for 1–3 min and passaged at a ratio of 1:3. The freezing medium consisted of 55% base medium, 40% FBS, and 5% DMSO.

### 2.2 CCK-8 cell viability assay

Logarithmically growing H9C2 cells were trypsinized, adjusted to a density of 5 × 10^3^ cells/well, and seeded in 96-well plates with 100 μL of medium per well. After 24 h of pre-incubation, cells were treated with different concentrations of CGA (50 μg/mL, 100 μg/mL, 200 μg/mL) for 24–48 h. Then, 10 μL of CCK-8 reagent (Thermo Fisher Scientific, PA137267) was added to each well, and the plates were incubated in the dark for 1–4 h. Absorbance at 450 nm (OD value) was measured using a microplate reader (Molecular Devices, China). Cell viability was calculated as (OD value of the experimental group - OD value of the blank group)/(OD value of the control group - OD value of the blank group) × 100%.

### 2.3 Live/dead staining

After removing the medium, cells were washed with PBS and stained with 2 μM acridine orange (AO) (labeling live cells, green fluorescence) and 1 μM propidium iodide (PI, labeling dead cells, red fluorescence) (Solarbio, China) for 15 min in the dark. The percentage of live cells was calculated by fluorescence microscopy.

### 2.4 SOD activity assay

Cell lysates were collected, and the ability of SOD (Beyotime, China) to scavenge superoxide anions was determined using a WST-8 kit, with activity expressed as U/mg protein.

### 2.5 Malondialdehyde (MDA) content detection

According to the operating procedures of the MDA assay kit (Beyotime, China), the thiobarbituric acid (TBA) method was used: Cell lysates were mixed with TBA reagent and heated, and the absorbance at 532 nm was measured. MDA content was expressed as nmol/mg protein.

### 2.6 Mitochondrial membrane potential detection (JC-1 probe method)

After treatment, cells were stained with 5 μg/mL JC-1 staining solution (Thermo Fisher Scientific, Waltham, MA, USA) for 20 min at 37 C in the dark. The ratio of red to green fluorescence was detected by flow cytometry (normal membrane potential forms red aggregates, while decreased membrane potential deaggregates into green monomers).

### 2.7 ATP content detection

Using a ATP assay kit (MedChemExpress, USA, HY-K0314): Cells were lysed, centrifuged to obtain the supernatant, mixed with luciferase reaction solution, and the luminescence intensity was detected by a microplate reader. ATP content was expressed as nmol/mg protein.

### 2.8 PINK1/parkin immunofluorescence (IF) staining

In the cell treatment step, one group was treated with the mitochondrial autophagy inhibitor Mdivi-1 (Aladdin, China; 338,967-87-6), while the remaining group was treated normally. The treatment method was the same as before. Cells were fixed with 4% paraformaldehyde for 15 min, permeabilized with 0.1% Triton X-100 (Beyotime, China, ST795) for 10 min, and blocked with 5% BSA for 1 h. Primary antibodies (anti-PINK1 and anti-Parkin, diluted 1:200) (Proteintech Group, China, 23274-1-AP; Bioss, China, bsm-61396R) were incubated overnight at 4 °C, followed by Cy3/FITC-labeled secondary antibodies (diluted 1:500) (Abcam, USA) for 1 h in the dark. Nuclei were counterstained with DAPI (Abcam, USA) before mounting. A fluorescence microscope (Olympus, Japan) was used to observe mitochondrial-localized PINK1/Parkin co-localization signals, and ImageJ was used to quantify fluorescence intensity and co-localization coefficients.

### 2.9 Western blot

Following a 4-h co-treatment with 200 µM H_2_O_2_ and 200 μg/mL OLEU, cells were exposed to 100 nM bafilomycin A1 (BafA1) (Abcam, ab120497) for an additional 4 h; control groups received no treatment. After the treatment, both the cells and the supernatant were collected for analysis. Protein extraction was performed using RIPA lysis buffer, followed by heat-denaturation in Laemmli sample buffer at 95 °C for 5 min. The proteins were then resolved by electrophoresis on 12% SDS-polyacrylamide gels.The separated proteins were electrotransferred onto PVDF membranes. After blocking with 5% non-fat milk in TBST, the membranes underwent overnight primary incubation at 4 °C with antibodies specific for P62 (Abcam, ab91526; 1:1,000), LC3 (Biorbyt, orb1495189; 1:2,000), and Parkin (Abcam, ab77924; 1:1,000), all diluted in antibody buffer. Following five washes with TBST (5 min each), the membranes were treated with an HRP-conjugated goat anti-rabbit IgG (H + L) secondary antibody (Sera Care, 5220-0336; 1:5,000 in 5% skim milk/TBST) for 1 h at room temperature and washed again.Protein signal detection was carried out using an ECL substrate, with exposure times adjusted on-the-fly for optimal results. The developed and fixed film was then scanned, and the resulting band intensities were quantified by densitometry using ImageJ software (version 1.53).

### 2.10 Quantitative assessment of mitophagic flux using MT-Keima

To quantitatively measure mitophagic flux, we employed the MT-Keima probe in conjunction with flow cytometry. The MT-Keima reagent (PPL, China, LV01230-2a) was reconstituted as directed by the manufacturer. Cardiomyocytes in the logarithmic growth phase were collected, centrifuged at 1,000 *g* for 5 min, and washed twice with PBS. They were then incubated with serum-free diluted MT-Keima for 30–60 min at 37 C under 5% CO_2_. After incubation, the cells were washed three times with PBS, detached using trypsin, centrifuged again, and resuspended in PBS. The cell density was adjusted to 1–5 × 10^6^ cells/mL for analysis by flow cytometry via the FITC/PE channels, with at least 10,000 events captured under optimized voltage and threshold conditions.

### 2.11 Establishment and grouping of rat MI models

Animal experiments were approved by the Animal Ethics Committee of Jilin University (No. SY202503029). All animal handling and care were performed in accordance with the Guide for the Care and Use of Laboratory Animals by the National Institutes of Health. Sixteen male SD rats (200 ± 20g) underwent ligation of the proximal left anterior descending coronary artery to induce MI, and fractional shortening (FS), ejection fraction (EF), end-diastolic volume (EDV), and end-systolic volume (ESV) were measured, with FS < 30% indicating successful MI modeling. Rats were divided into Sham, MI, and CGA groups.

### 2.12 Tail vein injection

The CGA group received daily tail vein injections (Q 24 h) of 1.2 mL/kg of 200 μg/mL CGA for 28 days. The sham-operated and MI groups received saline injections at the same volume and frequency as the CGA group. A 27G needle was used to puncture the lower 1/5 of the tail vein at a 45° angle, with an injection rate ≤0.2 mL/min.

### 2.13 Measurement of plasma concentration-time profile of CGA following intravenous administration in Rats​

To quantify the systemic exposure of CGA under a 200 μg/mL intravenous dosing regimen, a weight-normalized pharmacokinetic simulation was established in rats. The intravenous bolus solution was set at a concentration of 200 μg/mL with an injection volume of 1.0 mL/kg, corresponding to a dose of 0.20 mg/kg. Each group consisted of N = 4 (reflecting individual variations among four rats). Blood sampling time points were: 0, 1, 2, 5, 10, 15, 20, 30, 45, 60, 90, and 120 min.

### 2.14 Hematoxylin and eosin (H&E) and masson staining

After 28 days of continuous tail vein injection, H&E and Masson stains (Beyotime, China) were used on frozen sections of MI areas to detect collagen deposition and fibrosis. For H&E staining, myocardial tissue sections (4 μm) were deparaffinized, hydrated, and stained with hematoxylin for 5 min to show nuclei. After rinsing, eosin was applied for 2 min to stain the cytoplasm. For Masson’s trichrome, sections were treated with Weigert’s iron hematoxylin, Biebrich scarlet-acid fuchsin, and aniline blue to distinguish collagen fibers (blue) from muscle fibers (red). Both staining procedures included dehydration and mounting steps. Five random fields per slide were examined under light microscopy (400×) by two blinded investigators to assess tissue morphology and fibrosis.

### 2.15 Echocardiographic assessment of rat cardiac function

After 28 days of continuous tail vein injection, echocardiography (Vevo2100, Visualsonic, USA) was used to evaluate left ventricular function in all rats. Rats were anesthetized with isoflurane, and M-mode, short-axis, and long-axis views of the heart were recorded using an M250 probe. EF, FS, left ventricular internal dimension systole (LVIDs) and EDV were measured.

### 2.16 Electron microscopy

Fresh myocardial tissue (<1 mm^3^) was quickly harvested after 28 days of tail vein injection, immediately fixed in pre-cooled 2.5% glutaraldehyde (prepared in 0.1 M phosphate buffer, pH 7.4) at 4 C for 2 h, post-fixed with 1% osmium tetroxide for 2 h, dehydrated with acetone, and embedded in epoxy resin. Ultra-thin sections (50–70 nm) were stained with uranyl acetate and lead citrate for electron microscopy observation (Hitachi TEM system, Japan) and identification of mitochondrial autophagosomes.

### 2.17 Immunohistochemical and immunofluorescence analysis

CD31 is a biomarker for myocardial endothelial cells, while α-smooth muscle actin (α-SMA) is a biomarker for myocardial stromal cells. CD31 and α-SMA staining (Abclonal, China, A19014; Bioss, China, bs-10196R) was used to study vessel density and myocardial fibrosisin infarcted heart tissue, while PINK1 (1:200) and Parkin (1:200) (Proteintech Group, China, 23274-1-AP; Bioss, China, bsm-61396R) were used to detect mitophagy. Tissue sections underwent antigen retrieval in citrate buffer after deparaffinization. Endogenous peroxidase was blocked before incubating with primary antibodies (CD31, α-SMA, PINK1 and Parkin) overnight at 4 C. HRP-conjugated secondary antibody was applied for 1 h at room temperature, followed by 3,3′-diaminobenzidine (DAB) development and hematoxylin counterstaining. Negative controls omitted primary antibodies. Staining intensity was quantified using ImageJ from five random 400× fields per sample, with results verified by two independent observers.

### 2.18 Western blot of PINK1 and parkin

To further demonstrate that CGA promotes mitophagy and activates the PINK1/Parkin pathway, we performed WB analysis of PINK1 and Parkin in the Sham, MI, and CGA-treated groups. The primary antibodies used were as follows: PINK1 (Proteintech Group, China, 23274-1-AP; dilution 1:1,000) and Parkin (Abcam, ab77924; dilution 1:1,000). The experimental procedure was consistent with the method described previously.

### 2.19 Metabolomics analysis

Myocardial tissue (<50 mg) from the infarction and border zones of three rats each from the MI and CGA groups was rapidly collected, snap-frozen in liquid nitrogen, and stored at −80 C. Methanol-water (4:1) or acetonitrile-methanol-water (2:2:1) biphasic extraction was performed, followed by ultrasonic disruption and centrifugation (12,000×g, 15 min, 4 C). The LC-MS/MS platform was used for detection, and XCMS or MetaboAnalyst was used for peak alignment, normalization (PQN method), and missing value imputation. Quality control (QC) samples were prepared by pooling sample extracts and used to evaluate the reproducibility of the sample processing procedure under identical conditions. Unsupervised principal component analysis (PCA) was performed using the prcomp statistics function in R (http://www.r-project.org). Data were scaled to unit variance prior to unsupervised PCA. Metabolites with a variable importance in projection (VIP) > 1 and a P-value <0.05 (Student’s t-test) were selected as significantly differential metabolites and visualized using volcano plots. The original relative levels of the identified differential metabolites were row-scaled by unit variance scaling and presented in a heatmap generated with R packages. Identified metabolites were annotated against the KEGG Compound database (http://www.kegg.jp/kegg/compound/), and the annotated metabolites were subsequently mapped to the KEGG Pathway database (http://www.kegg.jp/kegg/pathway.html). Differential metabolites involved in the top-ranked human metabolome database (HMDB) Primary pathways based on P-values were further annotated and visualized using the HMDB database.

### 2.20 Biocompatibility assessment

By using the HE staining method, the effects of the injected drug on the heart, liver, spleen, lungs, and kidneys were evaluated.

### 2.21 Statistical analysis

GraphPad Prism seven software was used for statistical analysis. All data are presented as mean ± standard deviation (SD). Student’s t-test was used to determine statistical significance when comparing two groups. For comparisons involving three or more groups, analysis of variance (ANOVA) with *post hoc* testing was used. Statistical significance was defined as ns, not significant, *P < 0.05, **P < 0.01, ***P < 0.001, ****P < 0.0001.

## 3 Results

### 3.1 Protective effect of CGA on cells

**FIGURE 1:**
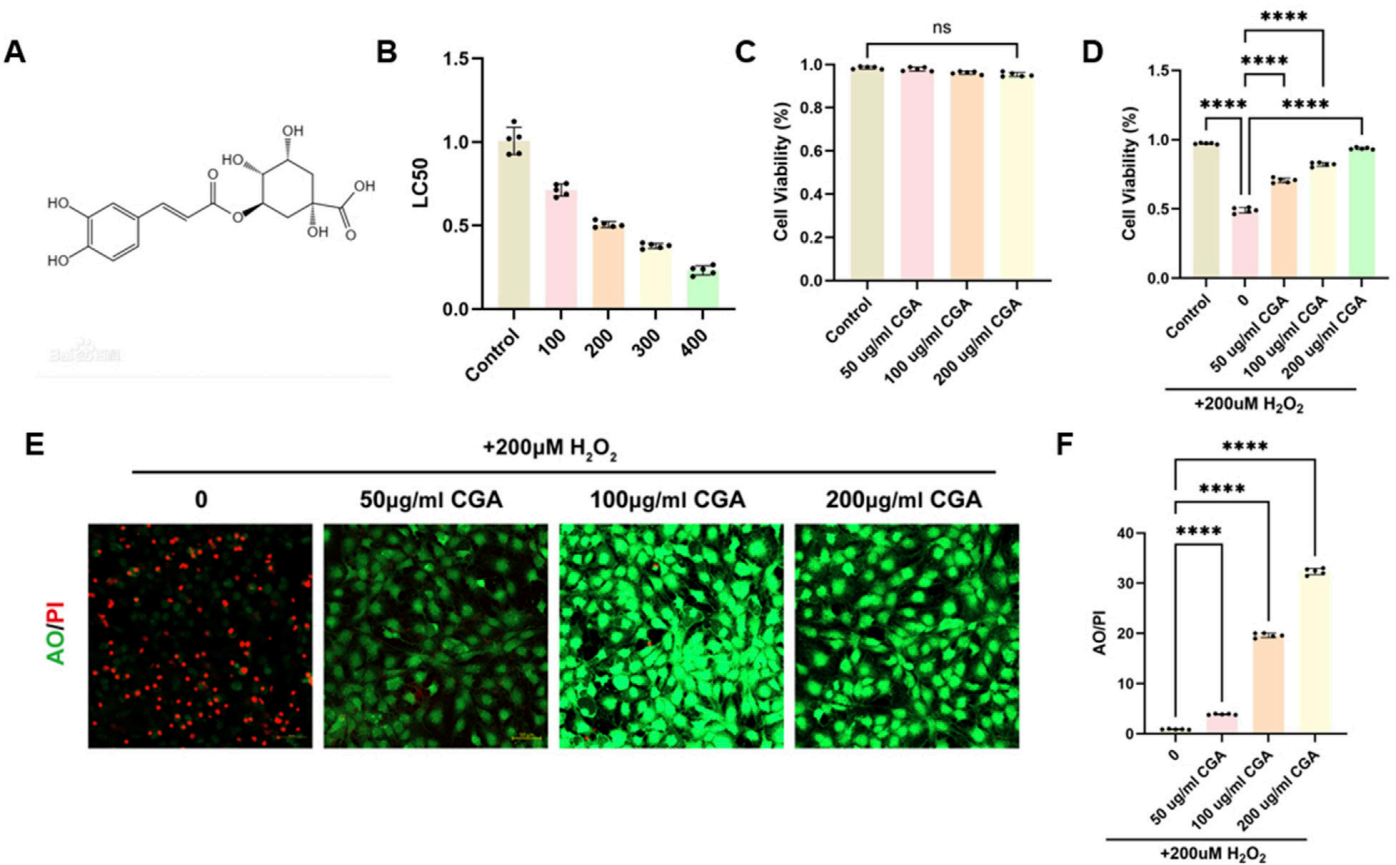
Effect of CGA on the viability of H9C2 cells. **(A)** Chemical structural formula of CGA. **(B)** Effect of different concentrations of CGA on cell survival rate. **(C)** Effect of different concentrations of H_2_O_2_ on cell survival rate. **(D)** The protective effect of CGA on H_2_O_2_-induced cell damage. **(E,F)** Acridine orange (AO)/Propidium Iodide (PI) staining and semi-quantitative fluorescence measurement. All the statistical data are represented as mean ± SD (n = 5 biologically independent samples for **(B‐F)** ns, not significant, ****P < 0.0001).

CGA is a condensed phenolic acid formed by caffeic acid and quinic acid, with the chemical formula C16H18O9 (Figure 1A) and CAS number 327-97-9. Supplementary Material confirms the biological properties of CGA (Desite, China, DST221010-021). The CCK assay demonstrates that CGA has no significant cytotoxicity on H9C2 cells within the range of 200 μg/mL (Figure 1B). The IC50 test revealed that 200 μM H2O2 was the most suitable concentration for this experiment (Figure 1C). CGA significantly improved cell viability, with 200 μg/mL CGA exhibiting the greatest effect (Figure 1D). Live/dead staining showed that CGA markedly reduced H2O2-induced cell death (Figures 1E,F).

### 3.2 Regulation of oxidative stress, SOD, and MDA

**FIGURE 2:**
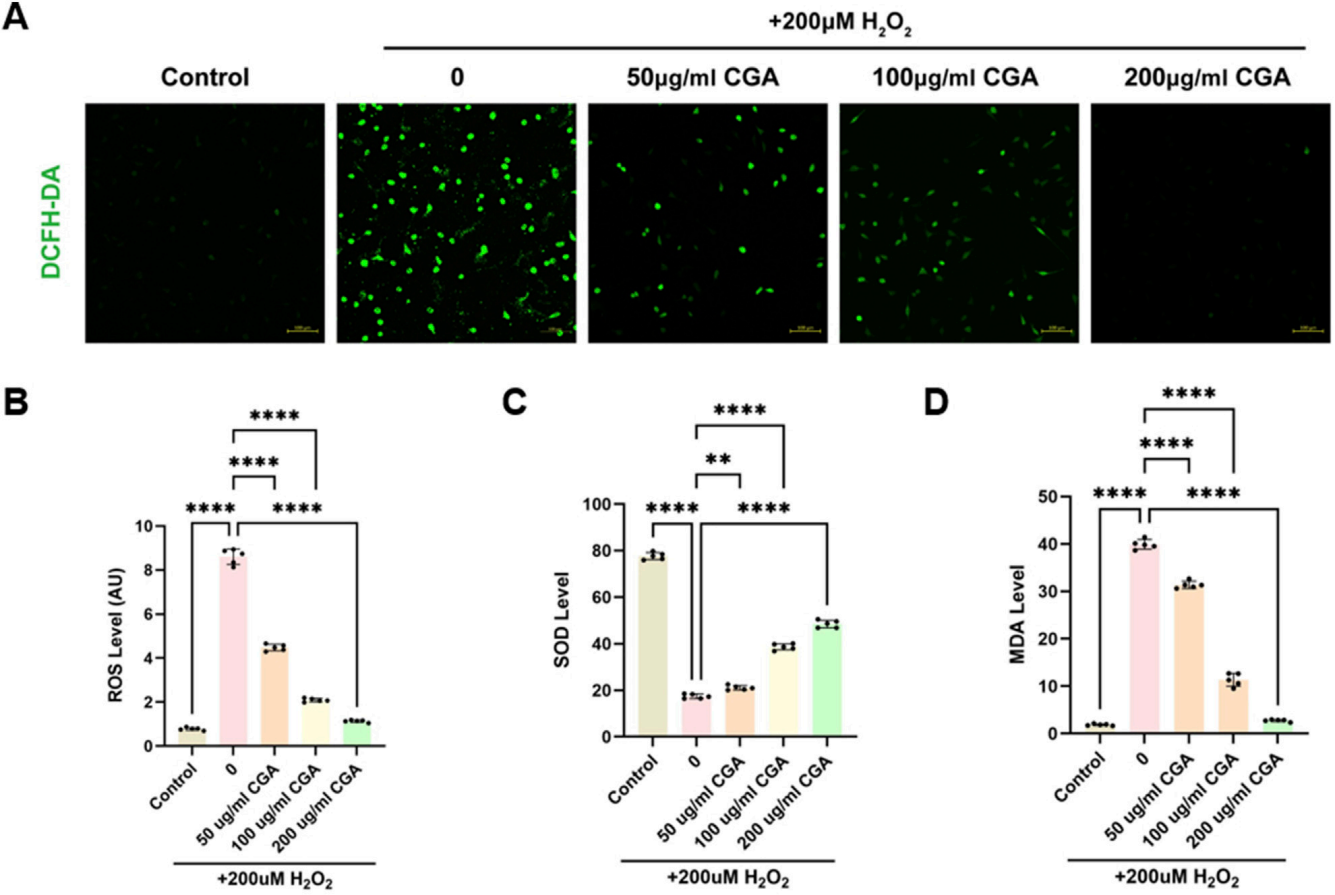
Measurement of oxidative stress, SOD, and MDA. **(A)** Fluorescence intensity images of cells stained with DCFH-DA after treatment with different concentrations of CGA. **(B)** ROS levels in cells treated with different concentrations of CGA. **(C)** Changes in SOD activity in cells after treatment with different concentrations of CGA. **(D)** MDA content in cells after treatment with different concentrations of CGA. All the statistical data are represented as mean ± SD (n = 5 biologically independent samples; **P < 0.01, ****P < 0.0001).

CGA significantly reduced ROS production induced by H2O2. Within the range of 200 μg/mL (Figures 2A,B), SOD levels in H9C2 cells increased significantly with increasing CGA concentrations (Figure 2C), while MDA levels decreased significantly (Figure 2D).

### 3.3 Regulation of mitochondrial energy metabolism

**FIGURE 3:**
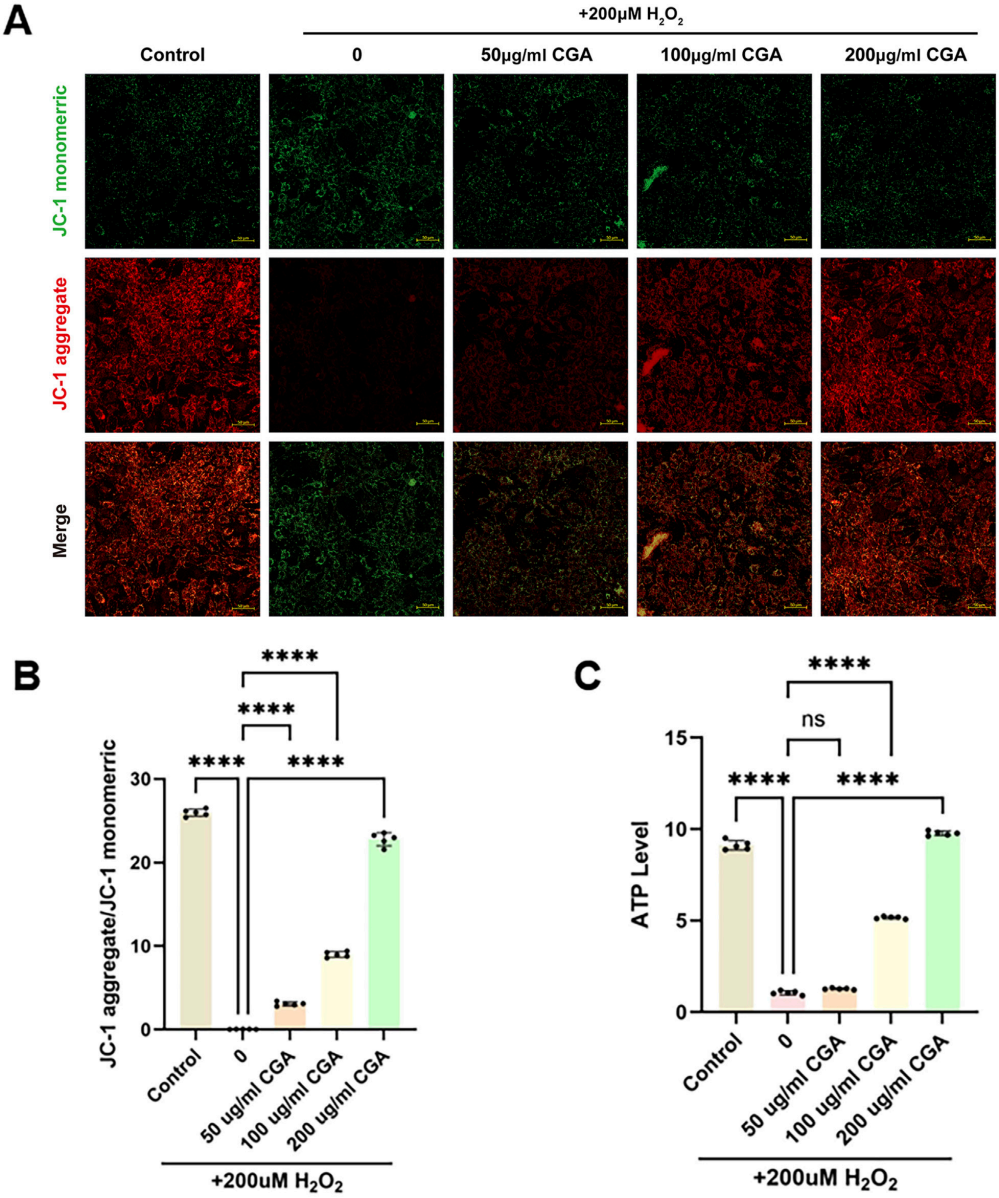
Measurement of mitochondrial membrane potential and ATP content. **(A,B)** Measurement of mitochondrial membrane potential using the JC-1 method and semi-quantitative fluorescence measurement. **(C)** Measurement of ATP content. All the statistical data are represented as mean ± SD (n = 5 biologically independent samples; ns, not significant, ****P < 0.0001).

CGA improved mitochondrial membrane potential levels in H9C2 cells (Figures 3A,B). H2O2 caused a decrease in ATP levels, which was significantly improved by 200 μg/mL CGA (Figure 3C).

### 3.4 Regulation of mitophagy by CGA

**FIGURE 4:**
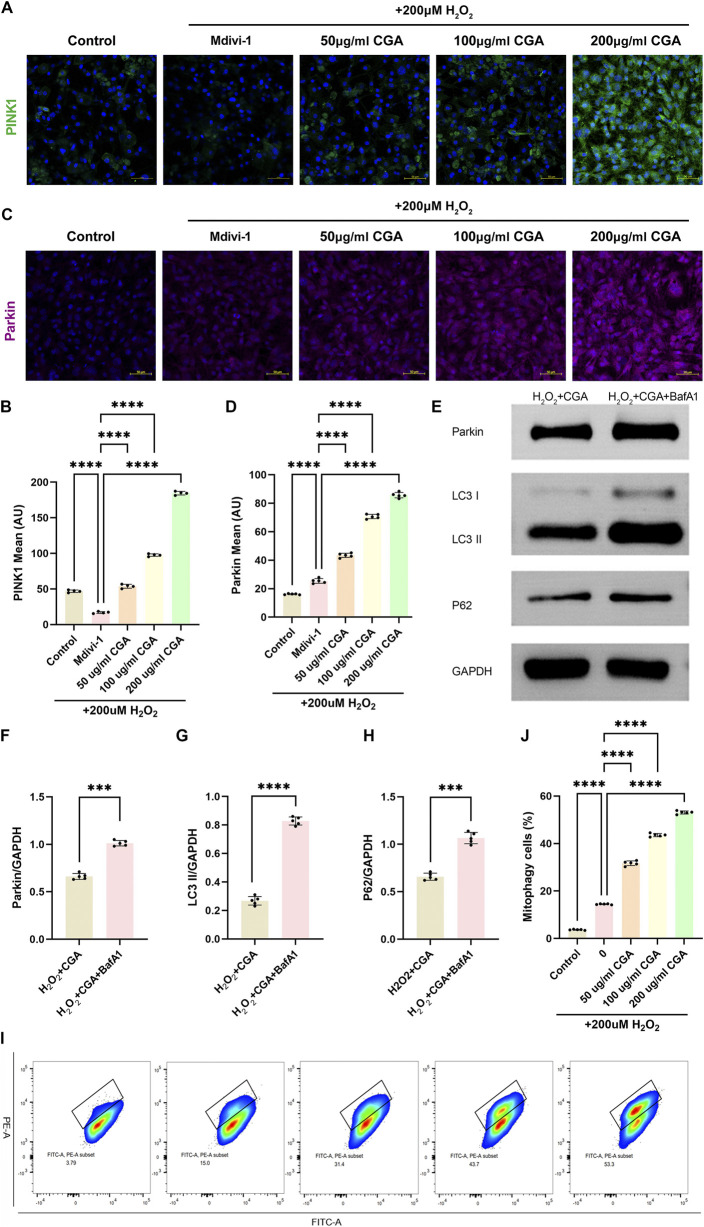
Activation of PINK1/Parkin-Mediated Mitophagy. **(A,B)** PINK1 immunofluorescence (IF) and semi-quantitative measurement. **(C,D)** Parkin IF and semi-quantitative measurement. **(E-H)** Western blot (WB) detection and quantification analysis of Parkin, LC3 and P62. **(I-J)** Flow cytometric analysis of MT-Keima with semi-quantitative assessment. All the statistical data are represented as mean ± SD (n = 4 biologically independent samples for **(A,B)** n = 5 biologically independent samples for **(C-J)** ***P < 0.001, ****P < 0.0001).

CGA upregulated the expression levels of mitophagy markers PINK1 (Figures 4A,B) and Parkin (Figures 4C,D), which were inhibited by the mitophagy inhibitor Mdivi-1. The upregulation of PINK1 and Parkin was concentration-dependent within the range of 200 μg/mL CGA. To specifically inhibit mitophagic flux, the lysosomal inhibitor BafA1 was applied. The results showed that the BafA1-co-treated CGA group displayed markedly increased levels of Parkin, LC3-II, and P62 compared to the CGA-only group, further supporting the promotive role of CGA in mitophagy (Figures 4E–H). Additionally, quantitative evaluation of mitophagic flux using the MT-Keima probe and flow cytometry indicated that CGA significantly enhanced mitophagy relative to cells exposed solely to H_2_O_2_ (Figures 4I,J).

### 3.5 Pharmacokinetic profile of intravenously administered CGA in rats

**FIGURE 5:**
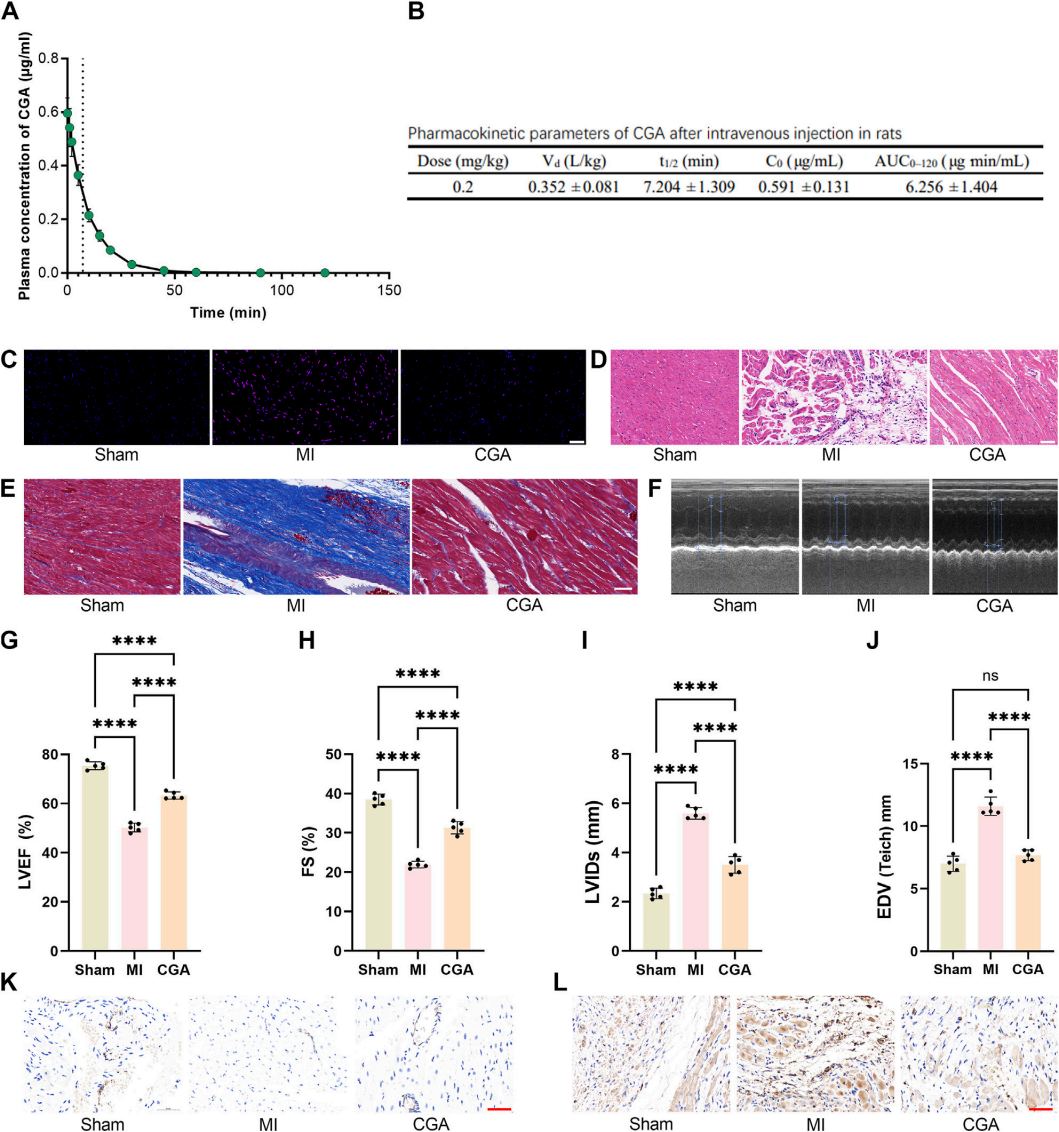
Effect of CGA on the rat MI model. **(A)** Plasma concentration-time curve of CGA after intravenous administration in Rats. The curve represents the mean plasma concentration at each time point, with the light-colored band representing ±1 SD. **(B)** Pharmacokinetic parameters of CGA after intravenous administration in Rats. **(C)** DHE staining of rat myocardial tissue. **(D)** HE staining. **(E)** Masson trichrome staining. **(F‐J)** comparison of echocardiograms and related indicators. **(K)** changes in CD31 expression in different treatment groups. **(L)** α-SMA immunohistochemical staining images. Scale bar, 50um. All the statistical data are represented as mean ± SD (n = 4 rats per group for **(A,B)** n = 5 biologically independent samples for **(C-I)** ns, not significant, ****P < 0.0001).

Following intravenous administration of a 200 μg/mL CGA solution (1.0 mL/kg, dose 0.20 mg/kg), the plasma drug concentration peaked immediately, with an average initial concentration of approximately 0.600 μg/mL. The plasma concentration then declined rapidly, dropping to around 0.220 μg/mL and 0.140 μg/mL at 10 and 15 min, respectively. By 30–45 min, concentrations approached the lower limit of quantification, and became largely undetectable after 60 min. The overall elimination process followed a one-compartment first-order decay model, with an estimated plasma half-life of approximately 7–8 min (Figure 5A). Figure 5B summarizes the pharmacokinetic parameters obtained under the 200 μg/mL solution and 1.0 mL/kg injection volume (dose 0.20 mg/kg) condition. The volume of distribution (V_d_) was 0.352 ± 0.081 L/kg, the corresponding initial concentration (C_0_) was 0.591 ± 0.131 μg/mL, and the individual half-life (t_1/2_) was 7.204 ± 1.309 min. The exposure measured as AUC_0–120_ was 6.256 ± 1.404 μg min/mL.

### 3.6 Effect of CGA on the rat MI model

CGA significantly reduced ROS levels in rats with MI (Figure 5C). H&E staining showed improved myocardial tissue after CGA treatment (Figure 5D), and Masson staining revealed a reduced area of myocardial fibrosis (Figure 5E). Echocardiography showed significantly improved cardiac function in rats with MI (Figure 5F), with increased EF, FS, LVIDs, EDV (Figures 5G–J). Immunohistochemical staining of CD31 and SMCs indicated that CGA promotes the expression of myocardial endothelial cells, reduces the expression of myocardial stromal cells, and inhibits myocardial fibrosis and remodeling (Figures 5I–K).

### 3.7 Regulation of mitophagy by CGA in rat MI models

**FIGURE 6:**
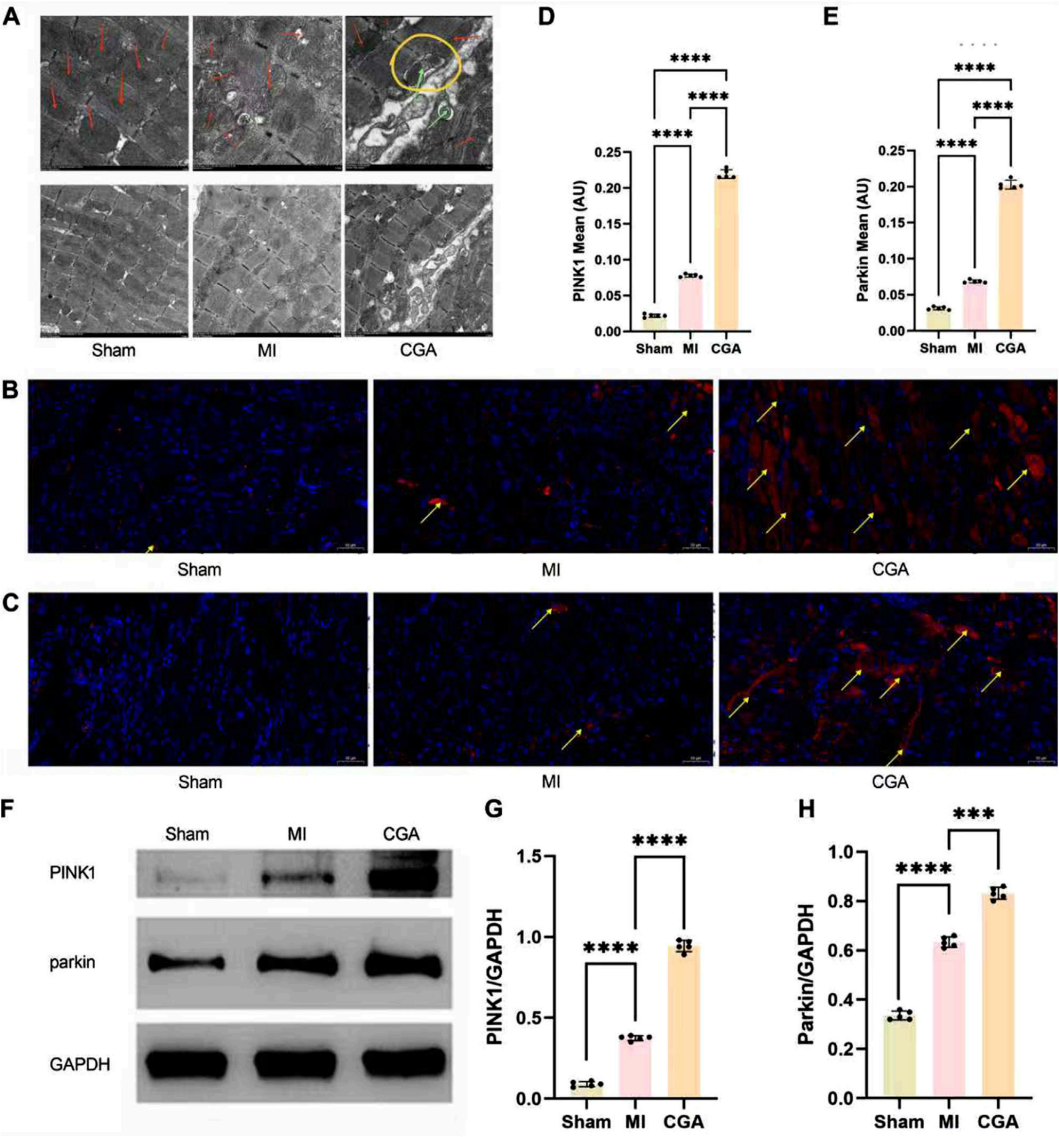
Regulation of CGA on mitophagy. **(A)** Electron microscopy images of myocardial tissue (6K × , 3K×). Red arrows indicate mitochondria, green arrows mark the occurrence of mitophagy, and yellow circles are used to highlight the process of mitophagy. **(B)** IF staining images of PINK1. The yellow arrows indicate PINK1-positive signals in the IF staining. **(C)** IF staining images of Parkin. The yellow arrows indicate Parkin-positive signals in the IF staining. **(D)** Semi-quantitative measurement of PINK1 IF staining. **(E)** Semi-quantitative measurement of Parkin IF staining. **(F‐H)** WB detection and quantification analysis of PINK1 and Parkin. Scale bar, 50um. All the statistical data are represented as mean ± SD (n = 5 rats per group; ***P < 0.001, ****P < 0.0001).

Electron microscopy showed that 200 μg/mL CGA significantly promoted mitophagy and repair of damaged mitochondria in rats (Figure 6A). During MI, the double-membrane structure of the mitochondria is disrupted. With the promotion of mitophagy by CGA, the mitochondrial structure was observed to be restored, accompanied by the emergence of numerous mitophagosomes. Within the yellow circles, typical “mitophagy” is visible, where mitochondria can be clearly identified within the double-membrane structures. Figures 6B–E shows the IF results demonstrating the upregulation of CGA on the expression levels of mitophagy markers PINK1 and Parkin in myocardial tissue. WB analysis further confirmed the regulatory effect of CGA on the expression of mitophagy markers PINK1 and Parkin in cardiac tissue (Figures 6F–H).

### 3.8 Metabolomics analysis

**FIGURE 7:**
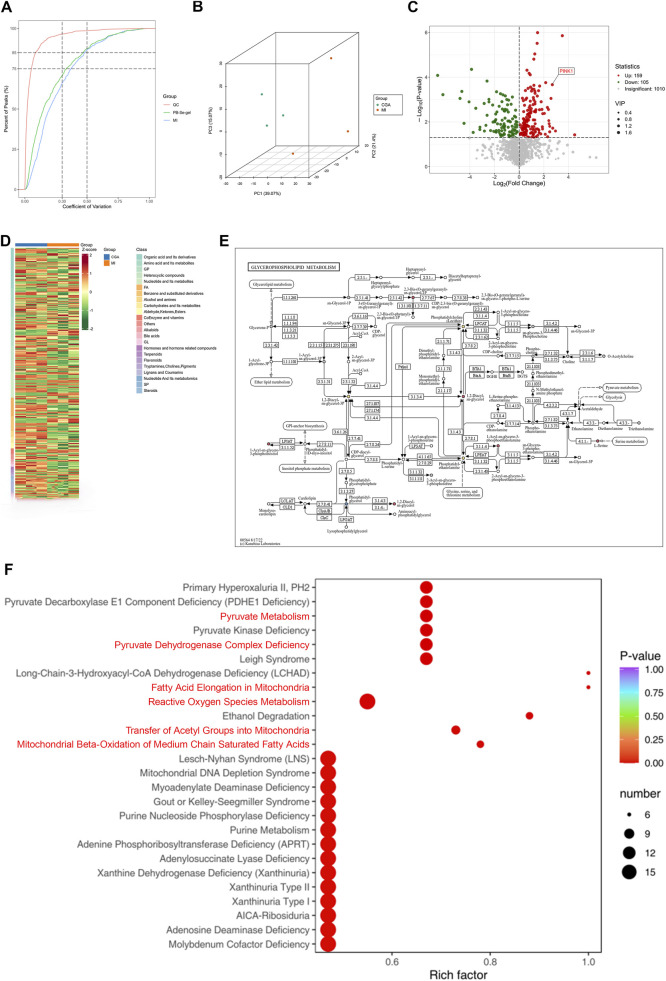
Metabolomics analysis. **(A)** Distribution of CV (coefficient of variation) across experimental groups. The X-axis represents CV values, while the Y-axis indicates the proportion of substances with CV values lower than the corresponding threshold relative to the total number of substances. Different colors correspond to sample groups, with QC denoting quality control samples. Two vertical reference lines (parallel to the y-axis) are drawn at CV values of 0.3 and 0.5, and two horizontal reference lines (parallel to the x-axis) indicate the thresholds where the cumulative proportion of substances reaches 75% and 85% of the total. **(B)** Principal Component Analysis (PCA) plot. PC1 and PC2 are the principal components, and different colored points represent different groups, with positional differences reflecting gene expression differences. **(C)** Differential gene expression heatmap. The heatmap reveals significantly differentially expressed genes between CGA and MI, with red and blue representing upregulation and downregulation, respectively, and the intensity of color reflecting the degree of expression change. The tree diagram on the left shows gene similarity. **(D)** Differential gene expression volcano plot: The horizontal axis represents the log2 fold change in gene expression, and the vertical axis represents the significance P value. Different colored points represent different groups, with positions reflecting significant changes in gene expression. **(E)** Glycerophospholipid metabolic pathway diagram. It illustrates the location and role of differentially expressed genes between CGA and MI in the glycerophospholipid metabolism pathway. Nodes represent genes, edges represent relationships, and color and size reflect expression changes. **(F)** HMDB Enrichment Plot of Differential Metabolites.​​ The x-axis represents the Rich factor corresponding to each pathway, while the y-axis displays pathway names (sorted by P-value). The color of the dots indicates the P-value, with redder shades denoting more significant enrichment. The size of the dots reflects the number of differential metabolites enriched in each pathway. (n = 3 rats per group).

The coefficient of variation (CV) is defined as the ratio of the standard deviation to the mean of the raw data, reflecting the degree of data dispersion. More than 85% of the metabolites in the quality control (QC) samples exhibited a CV value below 0.5, indicating relatively stable experimental data. The results presented in Figure 7A further confirm the reliability of the sequencing data. Non-targeted metabolomics analysis of the CGA and MI groups (n = 3) showed distinct metabolic profiles between the two groups (Figure 7B). PCA revealed that 159 metabolites were upregulated and 105 were downregulated in the CGA group compared to the MI group (p < 0.05) (Figure 7C). KEGG enrichment analysis showed that metabolic pathways were the most significantly enriched, with a Rich Factor close to 1.0, indicating their dominance in the samples. Additionally, the enrichment of nucleotide metabolism, thyroid hormone synthesis, and cofactor biosynthesis pathways suggests the reliability of the experimental results, as these pathways collectively regulate ROS levels and mitophagy through energy sensing, metabolic modulation, and redox balance (Figures 7D,E). HMDB enrichment analysis revealed that the following pathways—fatty acid elongation in mitochondria, mitochondrial beta-oxidation of medium chain saturated fatty acids, transfer of acetyl groups into mitochondria, pyruvate dehydrogenase complex deficiency, pyruvate metabolism, and reactive oxygen species metabolism—are all associated with the regulation of mitophagy and/or ROS. These results further confirm the regulatory role of CGA in ROS and mitophagy (Figure 7F).

### 3.9 Toxicity of CGA on rat liver, spleen, lungs, and kidneys

**FIGURE 8:**
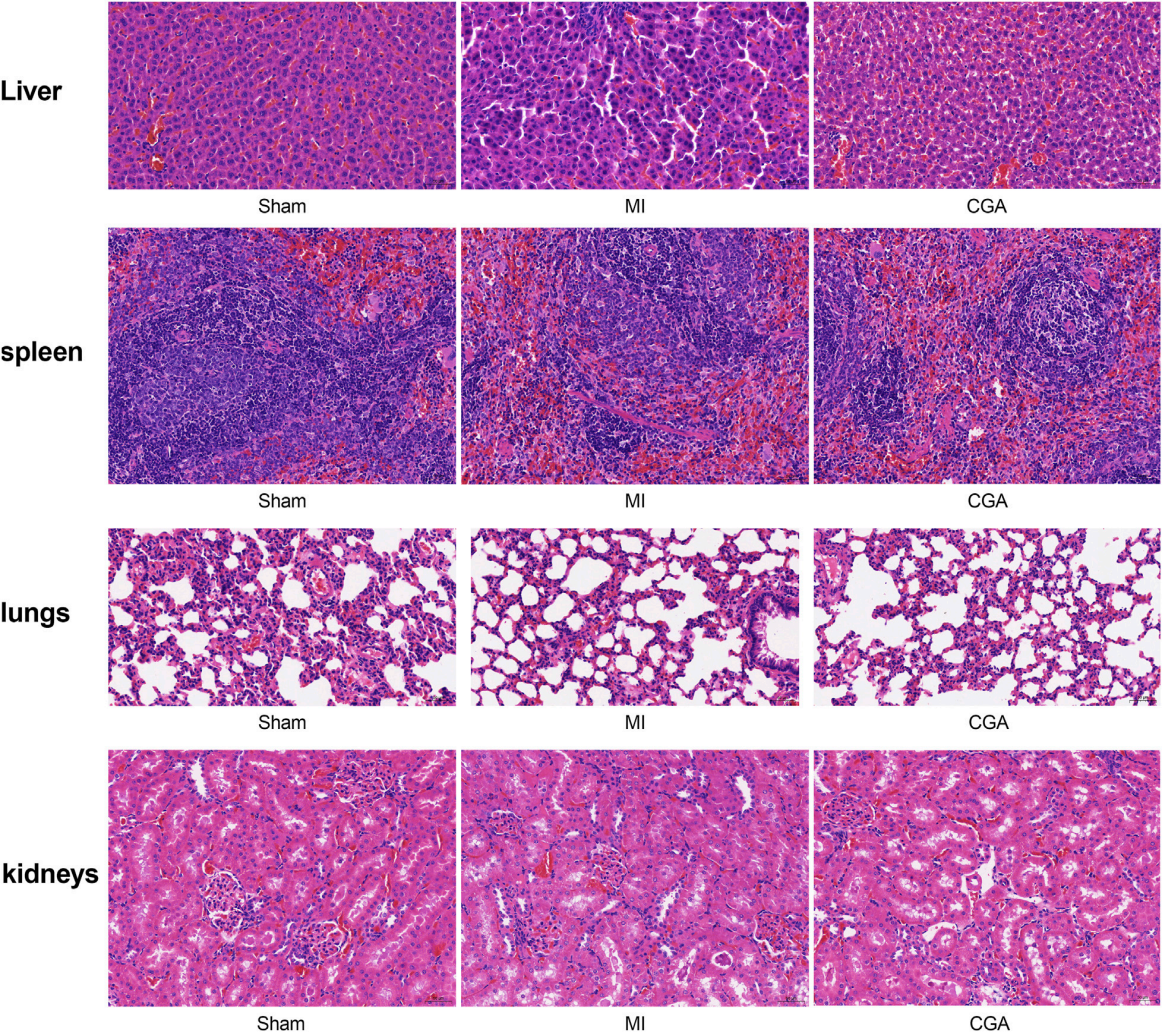
Exploration of CGA toxicity on various organs. Shows the HE staining of liver, spleen, lung, and kidney after different treatments. (n = 5 rats per group).

Immunohistochemical results showed no toxicity of 200 μg/mL CGA on rat liver, spleen, lungs, and kidneys (Figure 8).

## 4 Discussion

In this study, we systematically investigated the therapeutic potential of CGA in MI models, emphasizing its modulatory effects on mitochondrial damage and mitophagy through the PINK1/Parkin signaling pathway. Our findings not only provide new insights into the cardioprotective mechanisms of CGA but also highlight several innovative aspects of this research.

Key innovations of this study include:

1. Novel mechanistic insight into CGA’s cardioprotection

Our study innovatively explored the potential of CGA in promoting mitophagy in the context of MI. Unlike previous studies that have primarily focused on the antioxidant properties of CGA (Nguyen et al., 2024), we specifically examined its effects on the PINK1/Parkin pathway. We demonstrated that CGA induces a concentration-dependent upregulation of PINK1 and Parkin expression, thereby activating the PINK1/Parkin mitophagy pathway. Furthermore, we observed a significant enhancement of CGA-induced mitophagic flux through mt-Keima combined with flow cytometry. Moreover, the upregulation of Parkin, LC3-II, and p62 expression following lysosomal inhibitor treatment further confirmed the activation of mitophagy (Li et al., 2025). Our findings collectively demonstrate that CGA promotes the occurrence of mitophagy. This finding extends the understanding of CGA’s biological activities beyond its antioxidant effects and highlights its potential as a mitochondrial quality control agent (García-Niño et al., 2021; Lu et al., 2023).

2. Integration of mitochondrial function and mitophagy regulation

While mitochondrial dysfunction is known to contribute to MI (Wang et al., 2023), our study uniquely combined mitochondrial functional assays (membrane potential, ATP levels) with mitophagy markers (PINK1/Parkin expression) to establish a comprehensive picture of CGA’s effects. The Mdivi-1 inhibition experiment demonstrated the specificity of CGA’s regulation of mitophagy, providing a novel idea for the development of targeted myocardial protectants. The accumulation of Parkin, P62, and LC3-II upon lysosomal inhibition indicates disruption in autophagic degradation, confirming the activation of mitophagy upstream. The MT-Keima assay demonstrated that CGA enhances mitophagic flux. Electron microscopy results further validated the effectiveness of this mechanism *in vivo*. In addition to traditional echocardiographic assessments, we also performed histological staining, electron microscopy, and immunohistochemical analysis to provide multi-faceted evidence of CGA’s efficacy. Furthermore, we conducted metabolomics analysis to gain insights into the global metabolic changes induced by CGA treatment, which revealed its potential to modulate metabolic homeostasis in MI.

3. “Antioxidant-metabolism” dual pathway synergy

We also found that CGA achieves “antioxidant-energy metabolism” pathway synergy by simultaneously regulating SOD/MDA and ATP/MMP, providing a new paradigm for explaining the multi-target characteristics of polyphenolic compounds (Nie et al., 2025). The dose-dependent increase in SOD and reduction in MDA levels indicated that CGA activated the endogenous antioxidant system, consistent with the redox regulatory properties reported in neuronal models (Zheng et al., 2022). The CGA-induced upregulation of SOD may be mediated by the Nrf2/ARE pathway, which has been confirmed as a key target of phenolic acid compounds (Dai et al., 2024). Metabolomic analysis revealed that CGA treatment significantly affected the metabolic network, particularly organic acids and their derivatives, amino acids, and metabolites. KEGG enrichment analysis of differential metabolites showed that the metabolic pathways were the most significantly enriched, indicating their dominance in the samples. This is consistent with the regulation of mitochondrial ATP levels observed in this study. The significant upregulation of metabolites in the heatmap suggests that chlorogenic acid treatment may induce oxidative stress, prompting cells to enhance glutathione synthesis for reactive oxygen species (ROS) scavenging and redox balance maintenance. Additionally, the elevated levels of organic acids, nucleotides, and glycerophospholipids further indicate that chlorogenic acid increases metabolic demands to meet cellular energy requirements.

4. Clinical translation potential

Notably, this study achieved a breakthrough in the therapeutic time window. Animal experiments confirmed that CGA still exhibits significant efficacy in the late stage of MI (fibrosis stage). The negative toxicity results on the heart, liver, spleen, lungs, and kidneys, combined with the intravenous administration route, circumvent the common issue of low oral bioavailability of polyphenols (Veras et al., 2022). This approach not only ensured a more stable and consistent drug concentration but also allowed for more precise dose control, which is essential for evaluating the therapeutic effects of CGA in MI models (Veras et al., 2022). The safe dose of 200 μg/mL aligns with the latest EFSA tolerable intake level standard (400 mg/kg) (Bampidis et al., 2022), providing more possibilities for CGA’s clinical application.

Furthermore, although CGA is a natural polyphenol widely present in plant-based foods and has been reported to possess various pharmacological activities, its *in vivo* processes—particularly its pharmacokinetic characteristics—have been poorly studied. This research fills this knowledge gap. The fundamental principle of pharmacokinetics is to quantitatively describe the process of how the amount (or concentration) of a drug changes over time in the body through mathematical models and formulas, thereby predicting and optimizing dosing regimens to ensure that safe and effective concentrations are achieved and maintained at the site of action (Mager, 2025). As the results show, after intravenous administration, CGA exhibits an extremely rapid *in vivo* elimination process: its plasma half-life (t_1_/_2_) is only about 7–8 min, and plasma drug concentration becomes nearly undetectable 60 min after administration. The ultra-short plasma half-life indicates that CGA undergoes rapid and efficient metabolism and/or excretion in the body. This is consistent with the extensive metabolic characteristics of phenolic acid compounds (Chobot et al., 2013). A high clearance rate means the drug remains in the systemic circulation for a very short time. However, the duration of a drug’s pharmacological effects is not always perfectly synchronized with the concentration-time curve of the parent compound in plasma (Tibbitt et al., 2016). CGA may achieve long-lasting effects through the following mechanisms: CGA is rapidly metabolized in the body, and its metabolites (such as caffeic acid, ferulic acid, hippuric acid, etc.) may possess equal or even stronger biological activity. These metabolites may have a longer half-life or higher affinity for target binding, thereby becoming the true effective molecules. Thus, even though the parent drug disappears rapidly, its active metabolites can continue to exert pharmacological effects. Additionally, the drug may engage in brief but high-intensity interactions with targets, sufficient to trigger a downstream signaling cascade. Once this biological process is initiated, the continued presence of the drug is no longer necessary.

Despite the significant findings of this study, some limitations exist. For example, the upstream signaling pathway is not fully elucidated, the dose-response relationship above 200 μg/mL CGA lacks in-depth study, and validation in larger animals is required. Furthermore, in this study, we utilized male rats for the investigation, primarily based on the consideration that the stable long-term testosterone levels in males lead to smaller fluctuations in physiological indicators, thereby facilitating the standardization of experimental results (Beery and Zucker, 2011). However, existing literature suggests that there are no significant differences in experimental outcomes between male and female animals. Therefore, our findings can be generalized to the broader population (Guizzetti et al., 2016). In the future, we will continue to explore the mechanism of action of CGA to provide stronger support for its widespread clinical application. We will also pay increased attention to the results from female subjects to eliminate any potential influence of sex differences on the research findings. Moreover, during the current sequencing process, we utilized three samples per group. In future studies, we will further increase the sample size to obtain more precise results.

The pharmacodynamic results of CGA from this study also strongly suggest that to maintain its effective therapeutic concentration *in vivo*, it may be necessary to develop sustained-release or targeted delivery systems (e.g., nanoparticles, liposomes, transdermal patches, etc.) to counteract its rapid clearance, reduce dosing frequency, and improve patient compliance. Future pharmacodynamic studies must also focus on the activities of both CGA and its major metabolites. If the efficacy of CGA is indeed mediated by long-acting metabolites or mechanisms, then a once-daily or even less frequent dosing regimen would be feasible—though this must be verified through specific pharmacodynamic experiments. Pharmacokinetic studies should also be extended to these metabolites to comprehensively understand the material basis for its *in vivo* efficacy.

In conclusion, the present study highlights the innovative aspects of exploring CGA’s cardioprotective effects through modulating mitophagy via the PINK1/Parkin pathway, introducing a novel administration route, conducting comprehensive *in vivo* experiments, and emphasizing the safety of CGA. Tjhese findings not only expand our understanding of CGA’s therapeutic potential but also pave the way for future clinical trials investigating its application in MI treatment.

## Data Availability

The original contributions presented in the study are included in the article/Supplementary Material, further inquiries can be directed to the corresponding author.

## References

[B1] Ajoolabady A. Chiong M. Lavandero S. Klionsky D. J. Ren J. (2022). Mitophagy in cardiovascular diseases: molecular mechanisms, pathogenesis, and treatment. Trends Mol. Med. 28 (10), 836–849. 10.1016/j.molmed.2022.06.007 35879138 PMC9509460

[B2] Bampidis V. Azimonti G. Bastos M. L. Christensen H. Dusemund B. Fašmon Durjava M. (2022). Safety and efficacy of a feed additive consisting of guar gum for all animal species (A.I.P.G. Association for International Promotion of Gums). Efsa J. 20 (4), e07253. 10.2903/j.efsa.2022.7253 35505784 PMC9052196

[B3] Beery A. K. Zucker I. (2011). Sex bias in neuroscience and biomedical research. Neurosci. Biobehav Rev. 35 (3), 565–572. 10.1016/j.neubiorev.2010.07.002 20620164 PMC3008499

[B4] Bhimaraj A. Young J. B. (2024). Myocardial recovery. Methodist Debakey Cardiovasc J. 20 (4), 1–5. 10.14797/mdcvj.1446 39184163 PMC11342845

[B5] Cao Y. Chen X. Pan F. Wang M. Zhuang H. Chen J. (2023). Xinmaikang-mediated mitophagy attenuates atherosclerosis via the PINK1/Parkin signaling pathway. Phytomedicine 119, 154955. 10.1016/j.phymed.2023.154955 37572567

[B6] Chen L. Lv Y. Wu H. Wang Y. Xu Z. Liu G. (2024). Gastrodin exerts perioperative myocardial protection by improving mitophagy through the PINK1/Parkin pathway to reduce myocardial ischemia-reperfusion injury. Phytomedicine 133, 155900. 10.1016/j.phymed.2024.155900 39094441

[B7] Chobot V. Kubicova L. Bachmann G. Hadacek F. (2013). Versatile redox chemistry complicates antioxidant capacity assessment: flavonoids as milieu-dependent anti- and pro-oxidants. Int. J. Mol. Sci. 14 (6), 11830–11841. 10.3390/ijms140611830 23736691 PMC3709758

[B8] Dai T. Xiao Y. Zhang H. Shi Y. Wu F. (2024). Chlorogenic acid alleviates high glucose-induced HK-2 cell oxidative damage through activation of KEAP1/NRF2/ARE signaling pathway. Discov. Med. 36 (186), 1378–1385. 10.24976/Discov.Med.202436186.128 39054709

[B9] García-Niño W. R. Zazueta C. Buelna-Chontal M. Silva-Palacios A. (2021). Mitochondrial quality control in cardiac-conditioning strategies against ischemia-reperfusion injury. Life (Basel) 11 (11), 1123. 10.3390/life11111123 34832998 PMC8620839

[B10] Guizzetti M. Davies D. L. Egli M. Finn D. A. Molina P. Regunathan S. (2016). Sex and the lab: an alcohol-focused commentary on the NIH initiative to balance sex in cell and animal studies. Alcohol Clin. Exp. Res. 40 (6), 1182–1191. 10.1111/acer.13072 27154003 PMC4889570

[B11] Hu B. Li J. Gong D. Dai Y. Wang P. Wan L. (2022). Long-term consumption of food-derived chlorogenic acid protects mice against acetaminophen-induced hepatotoxicity via promoting PINK1-dependent mitophagy and inhibiting apoptosis. Toxics 10 (11), 665. 10.3390/toxics10110665 36355956 PMC9693533

[B12] Li J. Yang D. Li Z. Zhao M. Wang D. Sun Z. (2023). PINK1/Parkin-mediated mitophagy in neurodegenerative diseases. Ageing Res. Rev. 84, 101817. 10.1016/j.arr.2022.101817 36503124

[B13] Li L. Jin L. Tian Y. Wang J. (2025). Semaglutide enhances PINK1/Parkin-dependent mitophagy in hypoxia/reoxygenation-induced cardiomyocyte injury. Mol. Med. Rep. 31 (5), 111. 10.3892/mmr.2025.13476 40017118 PMC11884227

[B14] Liu Y. Li L. Wang Z. Zhang J. Zhou Z. (2023). Myocardial ischemia-reperfusion injury; Molecular mechanisms and prevention. Microvasc. Res. 149, 104565. 10.1016/j.mvr.2023.104565 37307911

[B15] Lu H. Tian Z. Cui Y. Liu Z. Ma X. (2020). Chlorogenic acid: a comprehensive review of the dietary sources, processing effects, bioavailability, beneficial properties, mechanisms of action, and future directions. Compr. Rev. Food Sci. Food Saf. 19 (6), 3130–3158. 10.1111/1541-4337.12620 33337063

[B16] Lu Y. Li Z. Zhang S. Zhang T. Liu Y. Zhang L. (2023). Cellular mitophagy: mechanism, roles in diseases and small molecule pharmacological regulation. Theranostics 13 (2), 736–766. 10.7150/thno.79876 36632220 PMC9830443

[B17] Mager D. E. (2025). Breadth of pharmacology modeling: fundamentals of pharmacokinetics, pharmacodynamics, and mechanistic modeling. Handb. Exp. Pharmacol. 10.1007/164_2025_746 40323416

[B18] Narendra D. P. Youle R. J. (2024). The role of PINK1-Parkin in mitochondrial quality control. Nat. Cell Biol. 26 (10), 1639–1651. 10.1038/s41556-024-01513-9 39358449

[B19] Nguyen V. Taine E. G. Meng D. Cui T. Tan W. (2024). Chlorogenic acid: a systematic review on the biological functions, mechanistic actions, and therapeutic potentials. Nutrients 16 (7), 924. 10.3390/nu16070924 38612964 PMC11013850

[B20] Nie W. Zhao X. Zhang Y. Zeng C. Yang H. Liu B. (2025). Chlorogenic acid alleviates DNCB-induced atopic dermatitis by inhibiting the Akt1/NF-κB signaling pathway. Eur. J. Pharmacol. 998, 177534. 10.1016/j.ejphar.2025.177534 40118327

[B21] Ramachandra C. J. A. Hernandez-Resendiz S. Crespo-Avilan G. E. Lin Y. H. Hausenloy D. J. (2020). Mitochondria in acute myocardial infarction and cardioprotection. EBioMedicine 57, 102884. 10.1016/j.ebiom.2020.102884 32653860 PMC7355051

[B22] Singh A. K. Singla R. K. Pandey A. K. (2023). Chlorogenic acid: a dietary phenolic acid with promising pharmacotherapeutic potential. Curr. Med. Chem. 30 (34), 3905–3926. 10.2174/0929867329666220816154634 35975861

[B23] Tibbitt M. W. Dahlman J. E. Langer R. (2016). Emerging frontiers in drug delivery. J. Am. Chem. Soc. 138 (3), 704–717. 10.1021/jacs.5b09974 26741786

[B24] Veras K. S. Fachel F. N. S. de Araújo B. V. Teixeira H. F. Koester L. S. (2022). Oral pharmacokinetics of hydroxycinnamic acids: an updated review. Pharmaceutics 14 (12), 2663. 10.3390/pharmaceutics14122663 36559157 PMC9784852

[B25] Wang Q. Liu T. Koci M. Wang Y. Fu Y. Ma M. (2023). Chlorogenic acid alleviated AFB1-induced hepatotoxicity by regulating mitochondrial function, activating Nrf2/HO-1, and inhibiting noncanonical NF-κB signaling pathway. Antioxidants (Basel) 12 (12), 2027. 10.3390/antiox12122027 38136147 PMC10740517

[B26] Yu Y. Zhang Z. Chang C. (2022). Chlorogenic acid intake guidance: sources, health benefits, and safety. Asia Pac J. Clin. Nutr. 31 (4), 602–610. 10.6133/apjcn.202212_31(4).0003 36576278

[B27] Zheng Y. Li L. Chen B. Fang Y. Lin W. Zhang T. (2022). Chlorogenic acid exerts neuroprotective effect against hypoxia-ischemia brain injury in neonatal rats by activating Sirt1 to regulate the Nrf2-NF-κB signaling pathway. Cell Commun. Signal. 20 (1), 84. 10.1186/s12964-022-00860-0 35689269 PMC9185968

