# Supplementary material for: Chlorogenic acid modulates mitochondrial damage and mitophagy to repair injured myocardial tissue and cells
Source: Front Pharmacol. 2025 Oct 1;16:1658090. doi: 10.3389/fphar.2025.1658090 (PMC12520970; doi:10.3389/fphar.2025.1658090)
Supplement: Supplementary file 1 [file DataSheet1.pdf]

S1, Certificate of Analysis

Chlorogenic acid

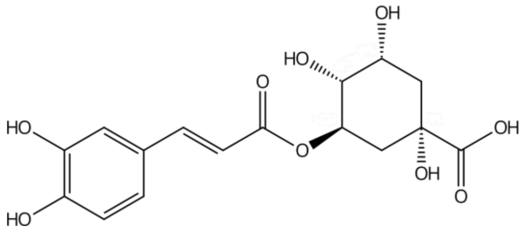

CAS Number: 327-97-9
Batch No.: DST221010-021
(M. F.: $C_{16}H_{18}O_9$
M. W.: 354.31

**Test Item**	**Specifications**	**Results**
Appearance	Off-White powder	Conforms
Purity (HPLC)	≥98.0%	99.01%

Test Method	Column:Ultimate XB-C18 4.6*250 mm,5μm; Column temperature: 35℃; Detection Mode:UV326 nm; Flow Rate:1.0ml/min; Sample dissolution:20%Methanol;Mobile Phase:A-Acetonitrile ,B-0.1% Phosphoric acid in water;Gradient elution:A,9%-9%,20min,9%-90%,5min.
Storage	Keep Out of light, dry and stored at 2-8℃.
Hint	If you encounter quality problems, please contact us within 15 days after receiving the products and thank you for your purchase.
Note	The product is only used for scientific research experiments and should not be used for human injection, food or other purposes. This solution must be freshly prepared before using to avoid degradation of the purity of the product and affect the experimental results.

HPLC

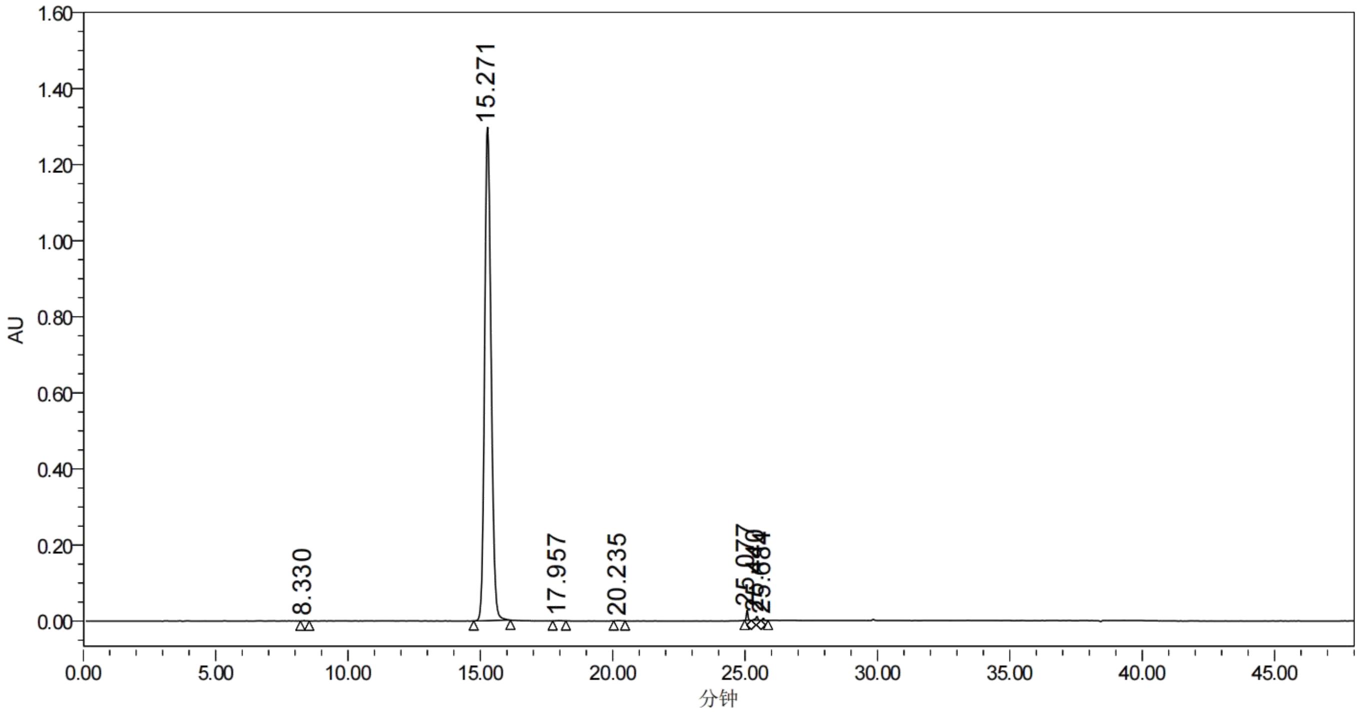

Peak Result

Serial Number	Retention Time (minutes)	Area (microvolts*seconds)	Height (microvolts)	Area Percentage
1	8.330	5776	569	0.03
2	15.271	21,991,783	1,297,366	99.01
3	17.957	9992	597	0.04
4	20.235	7195	492	0.03
5	25.077	111,308	25774	0.50
6	25.440	62,833	9626	0.28
7	25.684	22,290	5202	0.10
